# eHealth, digital information and technology use of men with prostate cancer

**DOI:** 10.1177/20552076241309214

**Published:** 2025-01-09

**Authors:** Stuart R Jackson, Paul Yu, Steven Sowter, Stefano Occhipinti, Suzanne Chambers, Scott Leslie, Manish I Patel

**Affiliations:** 1 4334 The University of Sydney, Camperdown, Australia; 2 Department of Urology, 570074 Westmead Hospital, University of Sydney, Sydney, Australia; 3 University of New South Wales, Wagga Wagga, Australia; 4 School of Applied Psychology, 5723 Griffith University, Brisbane, Australia; 5 Department of English and Communication, International Research Centre for the Advancement of Health Communication, 26680 Hong Kong Polytechnic University, Hong Kong, China; 6 276979 Faculty of Health Sciences, Australian Catholic University, Sydney, Australia; 7 Faculty of Health, 1994 University of Technology, Sydney, Australia; 8 2784 St Vincent's Health Network, Sydney, Australia; 9 RPA Institute of Academic Surgery, Royal Prince Alfred Hospital, Sydney, Australia; 10 Faculty of Health and Medicine, University of Sydney, Sydney, Australia

**Keywords:** Internet, eHealth, prostate, cancer, technology, information, digital, social

## Abstract

**Background:**

The investigation of digital information sources and technologies specifically used by men with prostate cancer is scarce. This study seeks to address current gaps in the literature by investigating prostate cancer–specific internet and technology use by men with prostate cancer and factors associated with this use.

**Methods:**

Cross-sectional surveys were conducted in three Australian urology clinics (local in Sydney, Western Sydney and Murrumbidgee) in 2023. Data analysis included descriptive and bivariate analysis. Chi square tests of independence, Mann–Whitney *U* tests and Fischer exact tests were used to assess demographic, prostate cancer-specific and psychometric variables with prostate cancer-specific usage of each website, social media and technology type.

**Results:**

A total of 349 men responded. Mean age of respondents was 69.6 years (SD 7.8). 74.5% (*n* = 260) had undergone radical prostatectomy, while 10% (*n* = 35) reported locally advanced/metastatic disease. Information websites were used by 77.7% (*n* = 271) of men. Social media was used by 37% (*n* = 129), and total internet use was 79.1% (*n* = 276). Younger age, higher education and higher income were commonly associated with a greater extent of use of information source and technology types. High variability in usage and factor association was demonstrated between and within analysed group categories.

**Conclusions:**

Men with prostate cancer use a broad variety of digital information sources and technologies to access prostate cancer information at a higher rate than ever before. This work stresses the significant variability in the extent of use which men demonstrate among these resources and the factors which may play a role in this behaviour.

## Introduction

Prostate cancer is the most diagnosed cancer in men worldwide, affecting >1.4 million men every year, and with over 375,000 deaths annually.^1^ Men diagnosed with prostate cancer commonly desire information to improve their understanding of the disease, to help select treatment options and for emotional and psychological support.^2^ In part, this is driven by the unique availability and suitability of several competing treatment modalities (e.g. surgery, radiotherapy, hormone therapy and active surveillance), which need to be balanced against patient needs and preferences.^2^ Information has been shown to enhance patients’ knowledge, decision-making and satisfaction with treatment/s, while also effecting the distress and anxiety, which are associated with a prostate cancer diagnosis and treatment.^3^ While research demonstrates similar information needs of prostate cancer populations across several developed nations, there is variation regarding reasons for information seeking, as well as volume and detail sought.^4^

Digital information accessed via eHealth communication technologies has become an attractive means of providing cost-effective and accessible tailored health information for men with prostate cancer.^5^ Accessibility and global usage rates of the internet have been increasing for some time in older populations, including men with prostate cancer.^6,7^ In Australia, a recent government report found that internet use has been spurred forward by COVID, rising from 71% to 98% among 65- to 74-year-olds, 2019–2022.^8^ Similarly, the proportion of those aged 75 and over who are online has almost doubled, from 52% to 94%.^8^

While digital usage rates are reaching higher highs, there are apprehensions regarding reliance on current information sources and technologies for men with prostate cancer.^9^ eHealth technologies necessitate consistent eHealth literacy improvement to be balanced with the natural cognitive and functional declines associated with ageing.^10^ Additionally, while there are little data exploring cancer-specific cognitive impairment effects on digital information engagement, the measurable cognitive processes that have been previously demonstrated with cancer diagnosis are associated with comprehension and higher-order functions necessary for information engagement.^11^ These cognitive concerns are compounded by other known gender differences in technology use and engagement, which have been previously purported to suggest that eHealth literacy or psychosocial differences of men (compared with women) also affect digital technology utilisation and uptake rates. Collectively, these aspects of aging, cancer diagnosis, cognition and sociology make local-level intervention selection, design and funding allocation difficult and costly for stakeholders of digital health and prostate cancer care.^12–14^

Despite concerns and duel concurrent growth of online information and communication technologies for health, our previous research has found that the investigation of online information sources and technologies related to men with prostate cancer are relatively scarce – several platforms are yet to be investigated thoroughly, or at all.^15^ Although several studies have demonstrated general and prostate cancer information-specific usage rates, most articles investigate either the internet as a singular information technology/media, or a specific platform, for example, Twitter (now ‘X’), or YouTube.^16^ As far as we are aware, for men with prostate cancer, no studies have aimed to investigate the use of the internet through a larger lens of its decentralised and diverse digital communication and technology parts.

We suggest that broad usage rates of the internet, website types, social media and technologies by this cohort necessitate further enquiry. This study seeks to address current gaps in the literature by investigating and demonstrating the broad and current prostate cancer-specific internet and technology use by men with prostate cancer. From a purposefully exploratory perspective, we aim to compare this use with a range of socio-demographic, psychometric and prostate cancer characteristics relevant to prostate cancer care. Gaining a broad understanding of these platforms’ use by men with prostate cancer will hold utility in assisting the design and implementation of digital health intervention strategies for this population, as well as directing future research endeavours into factors which may augment this use. We seek to answer the following questions:

- What online information sources (websites, social media, etc.) and technologies are used to access prostate cancer information by men with prostate cancer?
- What participant/prostate cancer characteristics of men with prostate cancer are associated with this use?

We hypothesise that socio-economic factors such as higher income, education and employment will be associated with increased likelihood of having used internet-based information sources and digital technologies. A second hypothesis is that prostate cancer characteristics of ‘alarm’ for patients – for example, high risk and active treatment status, advanced disease or active surveillance and biochemical failure, will be associated with higher use of these technologies due to distress and information seeking, whilst the presence of a peer navigator will also be associated with increased use. Finally, we hypothesise that decision regret, depression, anxiety, high self-efficacy and poor quality of life will result in increased use of these technologies and platforms.

## Methods

### Study design

This study was approved by the University of Sydney Human Ethics Research Committee (Project ID: 2022/617). This manuscript is the first presentation of data collected from ‘The PrE-Health Study’, a cross-sectional survey (modified Dillman survey methodology) and clinical data collection of Australian men with prostate cancer conducted in 2023. The PrE-Health Study is aimed at understanding the use of digital information and technology and eHealth use and literacy of men with prostate cancer. Due to the volume of data, this primary work presents baseline digital information and technology use of men with prostate cancer as the first analysis from the study.

### Setting and participants

All participants were over the age of 18, spoke English and had a biopsy-proven diagnosis of prostate cancer, which had undergone or begun management (e.g. prostatectomy, radiotherapy, active surveillance, watchful waiting, androgen deprivation therapy [ADT], focal therapy and chemotherapy).

Participants were recruited through clinics and digital advertisements. Clinic recruitment occurred through three private urology clinics in New South Wales, situated within the Local Health Districts of Sydney, Western Sydney and Murrumbidgee, respectively. These participants were recruited by clinic staff via use of clinic flyers, posters, letters sent via email/mail with participant information sheet and consent form, and referral to the same study materials by clinic staff during healthcare visits. Visual and text-based advertisements to social media websites and forums were also posted. This included Twitter (now ‘X’), Facebook (groups) and several online cancer support group forum websites. Potential participants were directed to research team contact details or an approved website with preliminary screening survey. All potential participants were contacted by phone and provided an opportunity to discuss the study with a research coordinator prior to enrolment.

Participants were given the option of digital or mail-based survey and consent forms to reduce participation bias. Those electing digital participation completed surveys and consent forms via Qualtrics survey website versions of the package, delivered to a preferred email address. Mail-based participants were sent a package to their preferred mailing address, including an addressed and stamped return envelope, which was to be returned after the survey was completed. Regardless of participation format, each package included a cover letter, participant information sheet, consent form and survey. Each survey sent to participants had a unique participant ID number and did not include any identifying information. Participants had 2 months to respond to the survey with reminders (email or letter dependent on form of participation) sent after 1 month.

Participants of clinics provided additional consent for their clinic records to be reviewed by the research team and to allow extraction of clinical history, diagnostic and treatment-based data related to their prostate cancer care. For this paper, this included pathology and imaging results, treatments received and dates of treatment and/or disease progression. These data were sought to establish prostate cancer characteristic groups of interest utilised in the study (see below), while aiming to reduce the effects of participant recall bias and health/cancer literacy via the retrospective structure of the survey.

### Questionnaire

The survey items and included questionnaires (see Supplemental File 1 for non-validated items) used in this work were developed by a multidisciplinary team of urology clinicians, nurses and psychology and clinical academics experienced in survey design and prostate cancer care. The questionnaire is composed of both non-validated questionnaire and validated psychometric instruments. The survey underwent pilot testing by 30 men with prostate cancer (recruited first as part of the approved study). Minor changes to language, formatting and item response options were implemented because of this work.

Informed by our previous investigations, participants were asked about digital information sources and technologies that they have used (yes/no) to access prostate cancer information.^15^ This included information based websites (search engine, news website, health provider website, hospital website, government healthcare website, education/university website, cancer/charity website, pharmaceutical/device company website, medical/scientific journal website, online decision aid website or portal based website [login and access personal information/results]); social media (Facebook, YouTube, Twitter, Instagram, LinkedIn, Pinterest, Reddit, TikTok, Snapchat, Wikipedia, community blogs, online support groups, Facebook Messenger and WhatsApp); and digital technologies (computer, mobile phone browser, SMS/text messaging, tablet device browser, mobile and tablet applications, podcasts, email, virtual and augmented reality, computer/video games and teleconference technology.

Participants were asked demographic questions including age, relationship status, country of birth, postcode, education, income, employment and government support (e.g. pension or disability). Questions regarding prostate cancer included year of diagnosis and whether a second opinion had been sought prior to the first treatment. Participants were also asked if they had a peer navigator who helped them access or navigate internet-based information sources for their prostate cancer care.

Psychometric instruments included in the survey have been previously validated in cancer populations. All scales are either in the public domain, or under copyright, with relevant permissions sought and granted prior to use. The EORTC QLQ-30^17^ is designed to measure cancer patients’ quality of life via physical, psychological and social functions.^18,19^ The Decision Regret Scale (DRS)^20^ measures ‘distress or remorse after a healthcare decision’ and was asked in the context of our participants’ first treatment decision.^21^ The PHQ-9 is the depression module of the Patient Health Questionnaire, which allows measure and diagnosis of depression and its symptoms.^22^ The GAD-7 is a self-report anxiety measure, which can evaluate anxiety symptomology.^23^ The GSES^24^ is a self-reported measure of self-efficacy, which has been previously correlated with emotion, optimism, depression, stress, health complaints and anxiety.^25^

### Variables

All website, social media and technology use was included as dichotomous variables (yes/no). To contextualise men's overarching use of digital information and technology with pre-existing literature, variables assessing use (yes/no) of ‘group categories’ were also formed as follows:

- Information website category = use of any listed information website type (e.g. search engine, news website and healthcare provider website);
- Social media category = use of any listed social media type (e.g. Facebook, blogs, WhatsApp and online support group);
- Social media network category = use of any named social media network platform type (e.g. Facebook or YouTube; others such as online support groups, Wikipedia, blogs and messenger-specific platforms were excluded);
- Internet information category = use of any listed information website or social media type; and
- Digital technology category = use of any listed digital technology type.

Socio-demographic data and relevant prostate cancer treatment characteristics were largely dichotomised to form clinically relevant groups of interest (Table 1), with age, age at diagnosis, DRS and GSES as continuous variables. Socio-demographic variables include age, relationship status (with partner or without partner), country of birth (Australia or overseas), education (primary/secondary school or university/vocational education), employment (working or retired) and government pension/disability support (yes/no). Income (<AUD 50,000 or ≥AUD 50,000) was dichotomised based on an approximation of the median personal yearly income detailed by the most recent data release of the Australian Bureau of Statistics (ABS).^26^ Residence was dichotomised (major city or regional/remote) by cross-referencing participant postcodes with the ABS Remoteness Structure (Australian Statistical Geography Standard Edition 3).^27^

**Table 1.** Socio-demographic, psychometric and prostate cancer characteristics of study participants.

Characteristic	Count	Characteristic	Count
Age (*n* = 349), mean (SD) (survey) Relationship status (*n* = 349), *n* (%) (survey) • With partner • Without partner Country of birth (*n* = 349), *n* (%) (survey) • Australia • Overseas Residence (*n* = 349), *n* (%) (survey) • Major city • Regional/remote Education (*n* = 349), *n* (%) (survey) • Primary/secondary school • University/vocational college Income (*n* = 312), *n* (%) (survey) • <AUD 50,000 • ≥50,000 Employment (*n* = 349), *n* (%) (survey) • Working • Retired Government pension/disability support (*n* = 346), *n* (%) (survey) • Yes • No Age at PCx diagnosis (*n* = 349), mean (SD) (survey) EAU risk stratification (clinical stage) (*n* = 297), *n* (%) (rooms) • Low • Intermediate/high Second opinion from health practitioner (*n* = 349), *n* (%) (survey) • Yes • No	• 69.6 (7.8) • 307 (88) • 42 (12) • 267 (76.5) • 82 (23.5) • 239 (68.5) • 110 (31.5) • 87 (24.9) • 262 (75.1) • 84 (26.9) • 228 (73.1) • 157 (45) • 192 (55) • 82 (23.7) • 264 (76.3) • 65.4 (7.8) • 100 (33.7) • 197 (69.3) • 98 (28.1) • 251 (71.9)	First treatment (localized disease) (*n* = 331), *n* (%) (rooms) • Active surveillance • Active treatment (surgery, radiotherapy, ADT) Active surveillance failure (*n* = 103), *n* (%) (rooms) • Yes • No Biochemical failure (*n* = 349), *n* (%) (rooms) • Yes • No Locally advanced/metastatic at any time (*n* = 349), *n* (%) (rooms) • Yes • No Decision Regret Scale (*n* = 339), mean (SD) PHQ-9 scale (*n* = 346), mean (SD) • No symptoms (summative score < 5), *n* (%) • Mild (summative score 5–9), *n* (%) • Moderate (summative score 10–14), *n* (%) • Moderate–severe (summative score 15–19), *n* (%) • Severe (summative score > 19), *n* (%) GAD-7 scale (*n* = 346), mean (SD) • No symptoms (summative score < 4), *n* (%) • Mild (summative score 5–9), *n* (%) • Moderate (summative score 10–14), *n* (%) • Severe (summative score > 14), *n* (%) GSES scale (*n* = 346), mean (SD) Peer navigator for digital PCx information (*n* = 346), *n* (%) (survey) • Yes • No EORTC QLQ-C30 functional TCI met (*n* = 349), *n* (%) (survey) • Yes • No EORTC QLQ-C30 symptom TCI met (*n* = 349), *n* (%) (survey) • Yes • No	• 103 (31.1) • 228 (68.9) • 55 (53.4) • 48 (46.6) • 49 (14) • 300 (86) • 35 (10) • 314 (90) • 8.5 (15.6) • 2.7 (3.4) • 274 (79.2) • 53 (15.3) • 15 (4.3) • 3 (0.9) • 1 (0.3) • 2 (3.1) • 284 (82.1) • 53 (15.3) • 6 (1.7) • 3 (0.9) • 32.5 (4.2) • 110 (31.8) • 236 (68.2) • 103 (29.5) • 246 (70.5) • 215 (61.6) • 134 (38.4)

Prostate cancer groups included the following: age at diagnosis; whether a second opinion had been sought from a health practitioner (yes/no); first treatment type for localised disease (active surveillance, active treatment – surgery, radiotherapy, ADT); failure of active surveillance (yes/no); European Association of Urology (EAU) clinical risk stratification (low, intermediate or high); biochemical failure (yes/no; for participants with localised disease treated with curative intent, was defined as PSA ≥ 0.20 after radical prostatectomy; PSA ≥ 2.0 after external beam radiotherapy; or, initiation of ADT, salvage radiotherapy or salvage prostatectomy after local therapy with curative intent); presence of locally advanced/metastatic disease at any time (yes/no); and whether a peer-navigator for digital prostate cancer information had been used (yes/no).

Results of the EORTC QLQ-C30 functional or symptom domains were dichotomised to assess for whether a threshold of clinical importance (TCI) had been met (yes/no).^28^ EORTC QLQ-C30 TCI is clinical threshold scores which the EORTC Quality of Life group developed to allow clinicians to flag symptomatic or functional impacts for their patients that may require intervention. The PHQ-9 (symptoms of depression or no symptoms of depression) and GAD-7 (symptoms of anxiety or no symptoms of anxiety) were included as dichotomised variables.

### Statistical analysis

The sample size of this study is a result of the overarching PrE-Health Study, which sought a minimum of 300 participants as part of the greater cross-sectional survey and quantitative instrument validation work. Considering historical usage rates of the internet in populations of men with prostate cancer (65%)^7^ and knowledge of contemporary older population internet use in Australia (>95–98%),^8^ a minimum sample size of 345 was calculated (95% confidence level and 5% margin of error). This is based upon a conservative anticipated frequency of 65% when considering older population internet use in Australia and a yearly prostate cancer diagnostic incidence of 25,000.^29^

A descriptive analysis of collected data was undertaken. Prostate cancer–specific use rates are reported for each digital information source and technology type, as well as overarching categories. Chi square tests of independence were used to assess the association of dichotomised variables with prostate cancer-specific usage of each website, social media and technology. If assumptions for *χ*^2^ testing were not met, Fisher's exact test was employed. Due to the violation of independent Student *t*-test assumptions, Mann–Whitney *U* tests were used to assess continuous variable group differences, with distributions assessed by visual inspection.

## Results

### Sample characteristics

A total of 396 participants enrolled in the Pre-eHealth study. A total of 349 participants returned completed or partially completed surveys, achieving a response rate of 87.9%. There were no significant differences found between clinical prostate cancer characteristics of complete and partial responders of survey items included in our analysis (Supplemental Table 1). The age range of respondents was 36–88, with mean age of 69.55 (SD 7.8, and mean age of diagnosis 65.4 (SD 7.8; Table 1). 74.5% of participants reported having undergone radical prostatectomy, and 10% reported locally advanced or metastatic disease.

*Rates of information and technology use* – *“What online information sources (websites, social media, etc) and technologies are used to access prostate cancer information by men with prostate cancer?”*

Table 2 reports total usage rates of all information website, social media and technology types used by men with prostate cancer to access prostate cancer information. The most used information website types included internet search engines (67.9%, *n* = 237) and websites of health providers (36.4%, *n* = 127). The most common forms of social media used were Wikipedia (19.5%, *n* = 68) and YouTube (18.9%, *n* = 66). The most used digital technologies were computers (69.1%, *n* = 241) and mobile phones (internet browser; 42.4%, *n* = 148). Notably, several social media (*n* = 9) and technology (*n* = 5) types had five users or less, of which five (TikTok, Snapchat, mobile phone applications, augmented reality and games) had no users at all.

**Table 2.** Prostate cancer-specific use of eHealth information sources and technologies.

Information website category	Total use, *n*/349 (%)
Internet search engine	237 (67.9)
News	33 (9.5)
Health provider (e.g. urologist/general practitioner)	127 (36.4)
Hospital	36 (10.3)
Government healthcare	67 (19.2)
Education (e.g. university)	44 (12.6)
Cancer/charity	107 (30.7)
Pharmaceutical/device company	33 (9.5)
Medical/scientific journal	74 (21.2)
Online decision aid	22 (6.3)
Patient portal (e.g. medical results)	15 (4.3)

Social media category	Total use, *n*/349 (%)
Facebook (FB)	25 (7.2)
YouTube	66 (18.9)
Twitter (Now ‘X’)	1 (0.3)
Instagram	2 (0.6)
LinkedIn	3 (0.9)
Pinterest	1 (0.3)
Reddit	1 (0.3)
TikTok	0 (0)
Snapchat	0 (0)
Wikipedia	68 (19.5)
Community blogs	16 (4.6)
Online support group	39 (11.2)
FB Messenger	5 (1.4)
WhatsApp	4 (1.1)

Digital technology category	Total use, *n*/349 (%)
Computer (desktop/laptop)	241 (69.1)
Mobile phone (browser)	148 (42.4)
SMS/text messages	31 (8.9)
Tablet device (browser)	114 (32.7)
Mobile phone applications	0 (0)
Tablet device applications	21 (6)
Podcasts	16 (4.6)
Email	1 (0.3)
Virtual reality	1 (0.3)
Augmented reality	0 (0)
Games (e.g. computer-, video-)	0 (0)
Teleconference (e.g. Zoom)	10 (2.9)
Group categories	Total use, *n*/349 (%)
Information websites	271 (77.7)
Social media	129 (37)
Social media networks	78 (22.3)
Internet information sources	276 (79.1)
Digital technology	265 (75.9)

Regarding group categories (Table 2), information websites were used by 77.7% (*n* = 271) of men. Social media (all) was used by 37% (*n* = 129), while social media networks use was lower at 22.3% (*n* = 78). Considering internet information sources overall, the total use rate to access prostate cancer information was 79.1% (*n* = 276; Table 3). Listed digital technologies were used by 75.9% (*n* = 265) of men.

**Table 3.** Statistically significant associations by digital information and technology type.

Information source or technology	Association	
Internet search engine	• ^Age • Education • Income • Government Support	• Peer navigator • ^Age at diagnosis • Symptom TCI
Health provider websites (e.g. urologist or general practitioner)	• ^Age • Income • Second opinion	• ^Age at diagnosis • Symptom TCI
Hospital website	• Country of birth • Education	• Biochemical recurrence
Government healthcare website	• ^Age • Education • Income	• Employment • Government support • ^Age at diagnosis
Education website (e.g. school or university)	• ^Age • Country of birth • Education	• Income • Employment • PHQ-9
Cancer/charity organisation website	• ^Age • Education • Income	• Government support • Peer navigator • ^Age at diagnosis
Pharmaceutical/device company website	• Second opinion • ^Self-Efficacy	• ^Age at diagnosis
Medical/scientific journal website	• ^Age • Country of birth • Residence • Education	• Income • Government support • Second opinion • PHQ-9
Online decision aid	• Country of birth	
YouTube	• Country of birth • EAU Risk Stratification	• First treatment • Biochemical recurrence
Instagram	• *Biochemical Recurrence	
LinkedIn	• *Second opinion	
Wikipedia	• Country of birth • Income	• GAD7 • Symptom TCI
Online support group	• ^Age • Education • Peer navigator	• EAU risk stratification • Functional TCI
FB Messenger	• *Peer Navigator	• *Second opinion
WhatsApp	• *Peer Navigator	
Computer (desktop/laptop)	• ^Age • Education • Income	• Peer navigator • ^Age at diagnosis • Symptom TCI
Mobile phone (internet browser)	• ^Age • Country of birth • Income • Employment	• Government support • Peer navigator • ^Age at diagnosis • Second opinion
SMS/text messages	• Country of birth • Education • Peer navigator	• EAU risk stratification • First treatment • *Advanced/metastatic
Tablet device (internet browser)	• ^Age • Income • Government support	• Peer navigator • ^Age at diagnosis
Tablet device applications	• Education	• Income
Podcasts	• *Second opinion	
Email	• Country of birth • Education • Peer navigator	• Second opinion • First treatment • ^Decision regret
Teleconference	• *Peer navigator	• ^Self-efficacy

*Note*. χ^2^ unless indicated: *Fisher exact test; ^ Mann–Whitney *U* test.

*Associations of online information sources and technology types* – *“What participant/prostate cancer characteristics of men with prostate cancer are associated with this use”?*

All statistically significant associations are reported in Table 3. In alternate to the below written manuscript, Table 3 is categorised by type of information website, social media and technology. No statistically significant associations were demonstrated regarding relationship status or whether men had failed active surveillance. No significant associations were demonstrated with news websites, patient portals, Facebook, Pinterest, Reddit, TikTok, Snapchat, mobile phone applications, virtual/augmented reality or games.

### Socio-demographic characteristics

#### Age

Users demonstrated a statistically significant lower median age compared with non-users for the following information website categorisations: internet search engines (user median 69, non-user median 72, *U* = 16,881.500, *Z* = 4.106, *p* < 0.001); health provider websites (user median 69, non-user median 71, *U* = 15,981.500, *Z* = 2.011, *p* = 0.044); government healthcare websites (user median 66, non-user median 71, *U* = 11,918.000, *Z* = 3.332, *p* = 0.001); education websites (user median 68.5, non-user median 70, *U* = 7970.000, *Z* = 2.016, *p* = 0.044); websites of cancer organisations/charities (user median 67, non-user median 71, *U* = 15,870.000, *Z* = 3.367, *p* = 0.001); and websites of medical/scientific journals (user median 67.5, non-user median 71, *U* = 11,700.500, *Z* = 1.982, *p* = 0.047). Online support groups were the only social media categorisation with statistically significant lower median age of user (67) compared with non-user (70), *U* = 7388.500, *Z* = 2.265, *p* = 0.024. A statistically significant lower median age was also demonstrated for users of several technologies: computers (user median 70, non-user median 71, *U* = 15,795.500, *Z* = 3.196, *p* = 0.001); mobile phones (user median 67, non-user median 71, *U* = 18,562.500, *Z* = 3.964, *p* < 0.001); and tablets (user median 69, non-user median 71, *U* = 15,653.500, *Z* = 2.557, *p* = 0.011).

#### Country of birth and residence

There was a statistically significant association between birth overseas (vs birth in Australia) and higher likelihood of using information website categorisations including hospitals, χ^2^ (1, *n* = 349) = 5.291, *p* = 0.021; education institutions, χ^2^ (1, *n* = 349) = 4.638, *p* = 0.031; medical/scientific journals, χ^2^ (1, *n* = 349) = 7.078, *p* = 0.008; and online decision aids, χ^2^ (1, *n* = 349) = 6.299, *p* = 0.012. Significant associations and higher likelihood of use were also demonstrated if born overseas for social media and technology categorisations including YouTube, χ^2^ (1, *n* = 349) = 13.730, *p* ≤ 0.001; Wikipedia, χ^2^ (1, *n* = 349) = 8.272, *p* = 0.004; mobile phones, χ^2^ (1, *n* = 349) = 5.556, *p* = 0.018; and SMS, χ^2^ (1, *n* = 349) = 6.436, *p* = 0.011. The only significant result regarding residence indicated that men living in major cities were more likely to use medical/scientific journal information websites than men living in regional or remote areas, χ^2^ (1, *n* = 349) = 6.907, *p* = 0.009.

#### Education

For men with university/vocational qualifications, a statistically significant association was demonstrated, with higher likelihood of using the following information website categories compared with men with school level education: internet search engines, χ^2^ (1, *n* = 349) = 12.020, *p* = 0.001; hospital websites, χ^2^ (1, *n* = 349) = 8.050, *p* = 0.005; government healthcare organisations, χ^2^ (1, *n* = 349) = 9.291, *p* = 0.002; educational institutions, χ^2^ (1, *n* = 349) = 6.748, *p* = 0.009; cancer/charity organisations, χ^2^ (1, *n* = 349) = 6.739, *p* = 0.009; and medical/scientific journals, χ^2^ (1, *n* = 349) = 5.082, *p* = 0.024. Similarly, significant associations and higher likelihood of use were associated with a higher level of education for online support groups, χ^2^ (1, *n* = 349) = 6.970, *p* = 0.008, and several technologies: computers, χ^2^ (1, *n* = 349) = 14.199, *p* < 0.001; SMS, χ^2^ (1, *n* = 349) = 6.206, *p* = 0.013; tablet applications, χ^2^ (1, *n* = 349) = 4.856, *p* = 0.028; and email, χ^2^ (1, *n* = 349) = 4.006, *p* = 0.045.

#### Income, employment and government support

Men with a higher income demonstrated a statistically significant higher likelihood of using several information website categories (compared with men of lower income): internet search engines, χ^2^ (1, *n* = 349) = 9.681, *p* = 0.002; health providers, χ^2^ (1, *n* = 349) = 5.003, *p* = 0.025; government healthcare organisations, χ^2^ (1, *n* = 349) = 7.730, *p* = 0.005; educational institutions, χ^2^ (1, *n* = 349) = 4.506, *p* = 0.034; cancer/charities, χ^2^ (1, *n* = 349) = 9.395, *p* = 0.003; and medical/scientific journals, χ^2^ (1, *n* = 349) = 1.173, *p* = 0.041. Compared with men with lower income, these men were also more likely to use: Wikipedia, χ^2^ (1, *n* = 349) = 4.173, *p* = 0.041; computers, χ^2^ (1, *n* = 349) = 8.847, *p* = 0.003; mobile phones, χ^2^ (1, *n* = 349) = 15.629, *p* < 0.001; tablets, χ^2^ (1, *n* = 349) = 13.416, *p* < 0.001; and tablet applications, χ^2^ (1, *n* = 349) = 5.220, *p* = 0.022. Men who were retired were less likely to have used websites of educational institutions, χ^2^ (1, *n* = 349) = 4.048, *p* = 0.044, and mobile phones, χ^2^ (1, *n* = 349) = 9.859, *p* = 0.002, to access prostate cancer information. Comparatively, men who were on government support were less likely to use several information website categories: internet search engines, χ^2^ (1, *n* = 349) = 8.806, *p* = 0.003; government healthcare websites, χ^2^ (1, *n* = 349) = 6.046, *p* = 0.014; cancer/charity organisations, χ^2^ (1, *n* = 349) = 5.227, *p* = 0.022; and medical/scientific journals, χ^2^ (1, *n* = 349) = 4.063, *p* = 0.044. Similar likelihood of lower use was also demonstrated for mobiles, χ^2^ (1, *n* = 349) = 12.936, *p* < 0.001, and tablets, χ^2^ (1, *n* = 349) = 6.952, *p* = 0.008.

#### Peer navigator presence

Presence of a peer navigator increased usage likelihood of search engines, χ^2^ (1, *n* = 349) = 4.625, *p* = 0.032; cancer/charity websites, χ^2^ (1, *n* = 349) = 5.034, *p* = 0.025; online support groups, χ^2^ (1, *n* = 349) = 4.181, *p* = 0.041; computers, χ^2^ (1, *n* = 349) = 9.666, *p* = 0.002; SMS, χ^2^ (1, *n* = 349) = 8.341, *p* = 0.004; and email to access prostate cancer information, χ^2^ (1, *n* = 349) = 14.732, *p* < 0.001. An association was also demonstrated between peer navigator presence and use of social media messaging systems Facebook Messenger (*p* = 0.037) and WhatsApp (*p* = 0.010) and teleconference technologies (*p* = 0.002).

### Prostate cancer clinical characteristics

#### Age at diagnosis

A Mann–Whitney *U* test was run to determine if there were differences in age at diagnosis for users and non-users of information sources and technologies used to access prostate cancer information. Users demonstrated a statistically significant lower median age compared with non-users for the following information website categorisations: internet search engines (user median 69, non-user median 72, *U* = 16823.500, *Z* = 4.040, *p* < 0.001); websites of health providers (user median 69, non-user median 71, *U* = 16103.000, *Z* = 2.214, *p* = 0.027); government healthcare organisations (user median 66, non-user median 71, *U* = 12133.000, *Z* = 3.621, *p* < 0.001); websites of cancer/charity organisations (user median 67, non-user median 71, *U* = 15976.000, *Z* = 3.488, *p* < 0.001); and websites of pharmaceutical/device companies (user median 69, non-user median 70.5, *U* = 6542.500, *Z* = 2.411, *p* = 0.016). A statistically significant lower median age was also demonstrated for users of several technologies: computers (user median 70, non-user median 71, *U* = 15800.000, *Z* = 3.200, *p* = 0.001); mobile phones (user median 67, non-user median 71, *U* = 18326.000, *Z* = 3.710, *p* < 0.001); and tablets (user median 69, non-user median 71, *U* = 15593.500, *Z* = 2.489, *p* = 0.013).

### European Association of Urology risk stratification, first treatment, biochemical recurrence and advanced disease

Intermediate-/high-risk men were more likely to use YouTube, χ^2^ (1, *n* = 349) = 6.610, *p* = 0.010; online support groups, χ^2^ (1, *n* = 349) = 5.201, *p* = 0.023, and SMS to access prostate cancer information, χ^2^ (1, *n* = 349) = 5.201, *p* = 0.023. Compared with men who underwent active surveillance, men who underwent active treatment for localised disease were more likely to use YouTube, χ^2^ (1, *n* = 349) = 5.588, *p* = 0.018; SMS, χ^2^ (1, *n* = 349) = 6.864, *p* = 0.009; and email, χ^2^ (1, *n* = 349) = 6.301, *p* = 0.012. Men who experienced biochemical recurrence were more likely to use hospital websites, χ^2^ (1, *n* = 349) = 6.277, *p* = 0.012; and YouTube, χ^2^ (1, *n* = 349) = 5.089, *p* = 0.024. An association was demonstrated with advanced/metastatic disease status and use of SMS (*p* = 0.025).

### Psychometric measures

#### Decision regret

A Mann–Whitney *U* test was run to determine if there were differences in decision regret scores for users and non-users of information sources and technologies used to access prostate cancer information. Users (5) demonstrated a higher and statistically significant median decision regret score compared with non-users (0) of email, *U* = 5628.500, *Z* = −2.848, *p* = 0.004.

#### Depression and anxiety

Those men experiencing symptoms of depression were less likely to use websites of educational institutions, χ^2^ (1, *n* = 349) = 4.201, *p* = 0.040, or academic/scientific journals, χ^2^ (1, *n* = 349) = 5.710, *p* = 0.017, for prostate cancer information, compared with men without symptoms of depression. Men with symptoms of anxiety were more likely to use Wikipedia to access prostate cancer information, χ^2^ (1, *n* = 349) = 5.780, *p* = 0.016.

#### General self-efficacy

A Mann–Whitney *U* test was run to determine if there were differences in self-efficacy for users and non-users of information sources and technologies used to access prostate cancer information. Users (33) demonstrated a higher and statistically significant median self-efficacy score compared with non-users (32) of pharmaceutical/device company information websites, *U* = 3883.000, *Z* = −2.355, *p* = 0.019. Similarly, scores were higher for users (mean rank = 237.65) vs non-users (mean rank = 171.59) of teleconference technologies, *U* = 1038.500, *Z* = −2.067, *p* = 0.039.

#### Quality of life

Men meeting a quality-of-life fTCI were more likely to use online support groups to access prostate cancer information, χ^2^ (1, *n* = 349) = 4.182, *p* = 0.041. Comparatively, men meeting a quality-of-life sTCI were more likely to use: search engines, χ^2^ (1, *n* = 349) = 4.500, *p* = 0.034; websites of health providers, χ^2^ (1, *n* = 349) = 8.133, *p* = 0.004; Wikipedia, χ^2^ (1, *n* = 349) = 7.891, *p* = 0.005; and computers to access prostate cancer information, χ^2^ (1, *n* = 349) = 6.289, *p* = 0.031.

## Discussion

This study uniquely demonstrates broad use rates of digital information sources and technologies to access prostate cancer information by men with prostate cancer. The significance of this work is underscored by the variability of information source and technology use illustrated as and within our defined group categories. Based on our previous work, several of these individual source and technology types are first of kind data reported for any prostate cancer population globally.^15^ While exploratory in nature, our analysis of factors associated with each of these digital information source and technology types provides a unique arm of comparison to clinicians and researchers who engage with eHealth, or in patient education and digital communication as part of their practice.

By collectively analysing broad source components of the internet, this study demonstrated that 79.1% of respondents used the internet to access prostate cancer information. This usage rate is 22.1% higher than a 2019 Canadian registry study of men with prostate cancer (*n* = 1320), which reported a usage rate of 65%.^7^ The rate of use by participants in our study also surpasses a more recent analysis of a mixed cancer populations (51% female) which coincidently held a lower median age of 52.2.^30^ In this work by Bender et al., usage rate of the internet as a source of cancer information or support was measured at 73%. We offer several hypotheses for this difference: (a) the impact of COVID-19 increasing eHealth and technology utilisation rates globally since 2019^31^; (b) the relatively high proportion of university/vocationally educated and higher income men in our cohort, with both characteristics historically associated with higher use of digital information sources and technologies^7^; (c) locoregional differences, for example, limited access to urological services for some populations in Australia driving men with prostate cancer to online resources^32–34^; and/or (d) our methodology capturing a broader range of internet-based information sources and technologies used to access prostate cancer information.

While prostate cancer-specific use of online information in our cohort appears high, we note that this is significantly less than recent Australian measures of generalised (i.e. non-prostate cancer-specific) internet use (65–74-year-olds = 98%; ≥74-year-olds = 94%).^8^ This is a phenomenon that has been demonstrated in other analyses, with up to 16.8% difference demonstrated between general use of the internet, and specific use to access prostate cancer information.^7^ Previously, men with prostate cancer have reported a distrust of online information sources, which may partially explain this divergence.^35^ There is also a likely contribution of eHealth literacy, which is usually lower in older populations, and yet to be adequately assessed in men with prostate cancer.^6,36^ These factors mirror our own anecdotal experience with patients who report difficulty pertaining online prostate cancer information to their individualised clinical experience. To our knowledge, there is no study that explores the characteristics of men who change their usage behaviours regarding online prostate cancer information/technology vs general use of the internet. This cohort of men requires further investigation, as a subgroup who may benefit from tailored interventions of a digital and/or non-digital nature to improve treatment selection and psychological outcomes.

In this work, the number of associations explored and demonstrated between demographics, prostate cancer characteristics and psychometric measures are numerous by purposeful exploratory design. Several relationships support earlier literature and our own hypothesis regarding use of digital technologies and information-seeking behaviours, for example, where being a younger man, having higher education, or higher income are more often associated with using the internet or eHealth technologies.^7^ We note however that that these associations do not cut across the entire spectrum of internet information sources and technologies within our work. We counsel readers that a portion of this result is likely due to notably low usage rates for several of the website, social media and technology types investigated. Sources/technologies of higher use and longevity more consistently reflect the historical relationships of previous literature, for example, use of basic information websites and subcategorisations therein. However, we highlight that this is not the absolute case for all sources with established maturity, for example, YouTube, basic Hospital Websites, Wikipedia and Tablet Devices. It is possible that cognitive heuristics prevalent in our prostate cancer population may account for some of the unrecognised behaviour effect on information source selection and technological engagement demonstrated in our results.^37,38^ This is whereby an inability to engage in systematic processing results in deferral to heuristic cognitive processes such as the expert opinion heuristic.^37^ Due to this, we also wonder whether current multidomain models of eHealth literacy currently put forward in modern literature effectively represent the experience of men with prostate cancer (or cancer patients as a whole) when engaging with cancer information via transactionally communicative digital technologies.^36,39^ Further investigation is necessary to understand why these discrepancies exist between information and technology sources for men with prostate cancer.

For many information sources and technologies assessed, this is the first study to investigate the association with clinical prostate cancer characteristics and our included psychometrics. Notably, the associations of ‘age at diagnosis’ variable strengthen several of the findings demonstrated by the ‘age’ variable, reinforcing age-related effects on digital engagement in previous literature.^7^ Of particular interest worth highlighting, is that men of higher prostate cancer risk stratification or experiencing biochemical recurrence were more likely to use several of the included online resources or technologies. We first surmise that this may be related to their distress, though literature is mixed regarding the psychological impact that men experiencing these disease states endure.^40,41^ While we did not directly measure distress in our study, measures of depression and anxiety symptomology suggest that the emotional/psychological state of men may influence the way they interact with individual digital information sources and technologies. However, the relatively low psychological burden (depression, anxiety, decision regret) of our cohort ultimately does make this difficult to interpret. This avenue of enquiry necessitates further investigation due to the known impacts of acute stress and anxiety on neurophysiology, comprehension, memory and the cyclical reinforcement of health anxiety states.^42–44^

An alternate hypothesis for men with advanced disease stages turning to digital information may be surmised when considering our quality-of-life data, whereby men meeting criteria for a symptom-based TCI were more likely to access several (but not all) information source types and technologies. Classically, prostate cancer is considered an indolent disease.^45^ Men who have experienced biochemical failure and/or advanced prostate cancer disease states generally experience a lower quality of life, and this is largely attributed to undesirable symptoms of disease or advanced treatments.^46,47^ While our initial assumption was that men being told they have biochemical failure or advanced disease may prompt a distress response with digital information seeking, these behaviours may instead be in relation to higher evidentiary symptom burden which is already appreciated in literature.^47^ In conjunction with this we note that our hypotheses that psychological distress and ‘alarm’ for patients results in increased use of these technologies is only partially supported by the data, with the majority demonstrating little difference between comparative groups. On reflection, this relationship may similarly be in keeping with the previously detailed cognitive heuristic processes of prostate cancer populations during diagnosis and treatment.^37,38^

The proportion of participants born overseas in our study was not insignificant. This variable was included with the knowledge that this heterogeneous group of culturally and linguistically diverse (CALD) people are present in almost all nations, in varying proportion. In the case of Australia in particular, half of first or second-generation Australians are born overseas, and one in five speak English as their second language at home.^48^ There were a significant number of associations demonstrating increased use of digital information sources and technologies to access prostate cancer information, relative to Australian-born men. We suggest that this relatively higher likelihood of use may be driven by known barriers to CALD healthcare in Australia and beyond.^48^ While digital health technologies may admittedly present some barriers to these groups, the ability to facilitate greater health access to these men via health information in first language, an ability to learn from people of their own culture, and access to personalised information, is likely reflected in this data in some part.^49^ YouTube for example, which is more likely to be used by our overseas-born population, features over 80 different languages, which can facilitate such exchanges.^50^ Conversely, Australian-born men being less likely to use these technologies compared to their overseas counterparts may be a function of their greater ability to navigate the national health system without use of these technologies. More specific research is needed to focus on CALD and overseas-born prostate cancer populations to understand the ways which eHealth may improve care access and quality.

Finally, the known positive presence of a peer navigator appears to be reinforced in our work, increasing the likelihood that men with prostate cancer will demonstrate increased access to online support groups and prostate cancer information from cancer/charity-based websites.^51^ While we would advocate that almost all men would benefit from a peer health navigator, clinicians should encourage men with low eHealth literacy or experience to seek out a digital peer navigator for support during critical stages of their illness journey. This may include men of CALD backgrounds. While little data exists for peer navigators of CALD men with prostate cancer in the digital sphere of health information access, there is evidence to suggest that peer navigation of more generalised health-based systems and information sources can have positive effects in this subgroup.^52,53^

Strengths of this work include the large survey population sourced across metropolitan and regional centres, digital and paper-based response pathway aimed to reduce bias, high response rate and a purposefully broad and unique analysis that establishes a platform for modern prostate cancer-based eHealth research. Limitations of this work include the retrospective nature of the survey, with no limitation on participation with time since diagnosis and treatment. This may result in recall bias which could affect results; however, we elected for this design to capture a purposefully broad assessment of men with prostate cancer, and not only those with a recent diagnosis. There are no formal classifications available which guide information website, social media or technology type/category classifications; thus results may differ if a different form of categorisation was utilised. However, types and categories we included were determined through a combined use of bibliometric analysis data,^15^ inductive reasoning from wider literature, personal experience of digital information sources and technologies, and previous anecdotal patient discussions of digital information and technology use. Regretfully, one recent technology that was not explored in our survey was natural language processing artificial intelligence models such as ChatGPT. This is a new and popular digital information source that requires evaluation for quality, safety and reliability as a prostate cancer information resource for patients. Finally, the binary nature of questions on usage of digital sources and technologies in this study prevents discourse about variation in use behaviour such as frequency of access and time spent on each resource. Each is important to the development of health intervention strategies and requires additional investigation to build on this work.

## Conclusion

This study is a uniquely broad addition to the literature, demonstrating utilisation rates and associations for a significant collection of digital information sources and technologies used by men with prostate cancer. Findings demonstrate the ever-increasing utilisation of these technologies, which is occurring globally and corroborate several known associations in international literature. Broadly, younger age, higher education and higher income were associated with higher rates of digital information source and technology use, though with exceptions across the analysed digital landscape. The diversity of usage and relationships demonstrated in this study supports the assertion that the modern internet requires greater active consideration as a decentralised digital landscape of communication and media technologies when considering patient care and communication. This is emphasised strongly in prostate cancer care, which comprises a unique cohort of patients and a multitude of available treatment modalities, and whereby cancer diagnosis may impact the ability to effectively navigate the plethora of digital information resources available. Considering the internet as a singular eHealth media or technology may stymie intervention and research progress in nations and states where internet penetrance is already pervasive. We advocate that each technology and source listed in our work demands ongoing enquiry as to its role, function and appropriateness for delivery of prostate cancer information to stakeholders. Further research is necessary to explore the relationships demonstrated in this work, to understand the impact of eHealth literacy and cancer-impacted cognition on eHealth information and technology utilisation, and to characterise the subgroup of men who are avoidant of digital resources as part of prostate cancer self-education and communication.

## Supplemental Material

sj-docx-1-dhj-10.1177_20552076241309214 - Supplemental material for eHealth, digital information and technology use of men with prostate cancer Supplemental material, sj-docx-1-dhj-10.1177_20552076241309214 for eHealth, digital information and technology use of men with prostate cancer by Stuart R Jackson, Paul Yu, Steven Sowter, Stefano Occhipinti, Suzanne Chambers, Scott Leslie and Manish I Patel in DIGITAL HEALTH

sj-docx-2-dhj-10.1177_20552076241309214 - Supplemental material for eHealth, digital information and technology use of men with prostate cancer Supplemental material, sj-docx-2-dhj-10.1177_20552076241309214 for eHealth, digital information and technology use of men with prostate cancer by Stuart R Jackson, Paul Yu, Steven Sowter, Stefano Occhipinti, Suzanne Chambers, Scott Leslie and Manish I Patel in DIGITAL HEALTH
