# Supplementary material for: eHealth, digital information and technology use of men with prostate cancer
Source: Digit Health. 2025 Jan 9;11:20552076241309214. doi: 10.1177/20552076241309214 (PMC11719434; doi:10.1177/20552076241309214)
Supplement: sj-docx-1-dhj-10.1177_20552076241309214 - Supplemental material for eHealth, digital information and technology use of men with prostate cancer [file sj-docx-1-dhj-10.1177_20552076241309214.docx]

Supplementary File

Questions:

Sociodemographics

1. What is your date of birth? (Day/month/year)
   1. __ __ / __ __ / __ __ __ __
2. What is your country of birth?
   1. _______________________
3. What is your marital status? (choose one)
   1. Married
   2. Divorced
   3. Widowed
   4. Separated
   5. Single, never married
   6. De facto relationship (not married, and living with partner of >2 years)
4. What is your highest level of attained formal education? (Choose one)
   1. Did not complete school to year 12
   2. Completed school to year 12
   3. Diploma or TAFE certificate
   4. Undergraduate degree
   5. Postgraduate degree
5. What is your annual income bracket? (Choose one – income includes money from employment, investments and/or retirement savings)
   1. Less than $20,000
   2. $20,001 - $50,000
   3. $50,001 - $80,000
   4. $80,001 - $110,000
   5. $110,001 - $140,000
   6. $140,001 - $170,000
   7. $170,001 - $200,000
   8. >$200,001
   9. Prefer not to say
6. Are you currently employed? (Choose one)
   1. Yes – full time
   2. Yes - part-time/casual
   3. No – seeking work
   4. No – retired from workforce
7. Do you receive any of the following? (tick as many that apply)
   1. Government age pension
   2. Department of Veteran Affairs (DVA) pension
   3. Disability support pension
   4. ☐ Prefer not to say

Prostate Cancer

1. What year were you diagnosed with prostate cancer?
   1. ___________________
2. Did you seek a second opinion on treatment (i.e. seeing a second urologist, or second oncologist) from any of the following health care providers? (tick as many that apply)
3. Urologist
4. Radiation Oncologist
5. Prostate cancer nurse
6. General practitioner
7. None

Digital Sources

1. Which of the following did you use when seeking information about prostate cancer ***prior to your*** ***first treatment*** ? (e.g. prostatectomy, hormone therapy, or radiotherapy, etc)? (tick all that apply)

| Websites | Tick any that you used: |
| --- | --- |
| Internet Search engine (e.g. Google, Bing, Yahoo) |  |
| News website (e.g. news.com.au, 9news, ABC Australia, Daily Telegraph, The Age, Crikey) |  |
| Health care provider’s website (e.g. urologist, radiation oncologist, oncologist, GP) |  |
| Hospital website |  |
| Government website (e.g. healthdirect.gov.au, cancervic.org.au) |  |
| Education website (e.g. produced by a school, or university) |  |
| Cancer/charity organisation website (e.g. Cancer council, Urological Society of AUS&NZ) |  |
| Pharmaceutical/medical device company website |  |
| Medical/scientific Journal Website |  |
| Online prostate cancer decision aid |  |
| Patient portal (a website that gives patients access to their own personal health information) |  |
| Social Media | Tick any that you used: |
| Wikipedia |  |
| Blog |  |
| Podcast |  |
| Facebook |  |
| YouTube |  |
| Twitter |  |
| Instagram |  |
| LinkedIn |  |
| Pinterest |  |
| Reddit |  |
| TikTok |  |
| Snapchat |  |
| Online support group (where prostate cancer patients talk to each other in posts) |  |
| WhatsApp |  |
| Facebook Messenger |  |
| Other Technologies | Tick any that you used: |
| Computer (desktop or laptop) |  |
| Mobile internet browser |  |
| Tablet internet browser |  |
| Email (i.e. seeking information personally, or via a clinic bulletin on prostate cancer) |  |
| Mobile phone application/s |  |
| Tablet application/s |  |
| Web application (a program on the internet, accessed via a web browser using a log in) |  |
| Text message (via mobile) |  |
| Virtual reality (using technology to create a simulated environment) |  |
| Augmented reality (using technology to overlay digital information over the real environment) |  |
| Video/console/computer games |  |
| I did not use any of the above |  |

1. Which of the following have you used when seeking information about prostate cancer, ***­since*** your first treatment (tick all that apply)

| Websites | Tick any that you used: |
| --- | --- |
| Internet Search engine (e.g. Google, Bing, Yahoo) |  |
| News website (e.g. news.com.au, 9news, ABC Australia, Daily Telegraph, The Age, Crikey) |  |
| Health care provider’s website (e.g. urologist, radiation oncologist, oncologist, GP) |  |
| Hospital website |  |
| Government website (e.g. healthdirect.gov.au, cancervic.org.au) |  |
| Education website (e.g. produced by a school, or university) |  |
| Cancer/charity organisation website (e.g. Cancer council, Urological Society of AUS&NZ) |  |
| Pharmaceutical/medical device company website |  |
| Medical/scientific Journal Website |  |
| Online prostate cancer decision aid |  |
| Patient portal (a website that gives patients access to their own personal health information) |  |
| Social Media | Tick any that you used: |
| Wikipedia |  |
| Blog |  |
| Podcast |  |
| Facebook |  |
| YouTube |  |
| Twitter |  |
| Instagram |  |
| LinkedIn |  |
| Pinterest |  |
| Reddit |  |
| TikTok |  |
| Snapchat |  |
| Online support group (where prostate cancer patients talk to each other in posts) |  |
| WhatsApp |  |
| Facebook Messenger |  |
| Other Technologies | Tick any that you used: |
| Computer (desktop or laptop) |  |
| Mobile internet browser |  |
| Tablet internet browser |  |
| Email (i.e. seeking information personally, or via a clinic bulletin on prostate cancer) |  |
| Mobile phone application/s |  |
| Tablet application/s |  |
| Web application (a program on the internet, accessed via a web browser using a log in) |  |
| Text message (via mobile) |  |
| Virtual reality (using technology to create a simulated environment) |  |
| Augmented reality (using technology to overlay digital information over the real environment) |  |
| Video/console/computer games |  |
| I did not use any of the above |  |

1. Have any of these people helped you to access or navigate internet-based information sources (such as those mentioned in previous questions) for your prostate cancer care? (Tick all that apply)
   1. Partner (Husband/Wife/De Facto)
   2. Child
   3. Friend
   4. Parent
   5. Co-worker
   6. Other, if so, please specify ____________________________

Supplementary Tables

|  | **Participant (full response, relevant survey)**  **(n = 330)** | **Participant (partial response relevant survey; n = 19)** | **P-value** |
| --- | --- | --- | --- |
| Age at diagnosis   - ≤65 years of age, n (%) - >65 years of age, n (%) | - 167 - 163 | - 6 - 13 | .107 |
| Age (current)   - ≤65 years of age, n (%) - >65 years of age, n (%) | - 102 - 228 | - 2 - 17 | .059 |
| EAU Risk Group Classification   - Low - Intermediate-High | - 95 - 188 | - 5 - 9 | .537 |
| First Treatment (localised disease)   - Active Surveillance - Active Treatment | - 97 - 215 | - 6 - 13 | .964 |
| Failed Active Surveillance   - Yes - No | - 52 - 45 | - 3 - 3 | .594 |
| Biochemical Recurrence   - Yes - No | - 46 - 284 | - 3 - 16 | .738 |
| Locally Advanced/Metastatic Disease   - Yes - No | - 35 - 295 | - 0 - 19 | .237 |

Supplementary Table 1. Prostate Cancer Characteristics of full and partial respondents.
